# Case of Obstinate and Fatal Constipation

**Published:** 1847-12

**Authors:** 


### *C..se of Obstinate and Fatal Constipation.*

To the Editor of the Boston Medical and Surgical Journal.

Sir,—I take the liberty of sending you an account of a case, the most remarkable that has ever occurred in my practice; and if you deem it of any interest to your readers, you are at liberty to publish it in your journal. The case was one of obstinate and fatal constipation, from insidious inflammation.

The patient was a married lady of our city, in middle life, and between four and five months advanced in pregnancy. I was called to the case some two or three days after she was taken down with symptoms of what she and her friends called colic—similar attacks of which she had had frequently before, and they commenced treating her with laudanum in the usual way, but without any effect.

When I saw her there was no tenderness over the epigastrium upon pressure, and no fever—she simply had violent pain over the region of colon and stomach, attended with considerable flatulence, which would entirely disappear for a while and then return again. In a word, the case seemed to be clearly one of *flatulent colic.*—And as such, a solution of half a drachm of bi-carb. soda, with fifty drops of laudanum, was administered, to be followed with a full dose of oil, having the same amount of laudanum, provided the pain returned.

There was ease for two or three hours, when the pain returned and the oil and laudanum were given, but rejected. Cal and pul. Dov. *aa* gr. x, were given, with some relief for a short time. This was followed by another dose of oil, which was again rejected. The pain returning and the stomach irritable, the following prescription was ordered:—℞., S. mur. hydrag., gr. xij.; pulv. opii, gr. ij. M. Ft. pil. iv. One to be taken every two hours with laudannm, gtts. xi., between the pills, if they should prove insufficient to quiet pain. The pills and laudanum were all taken with but partial relief. The stomach being somewhat irritable, a dose of magnesia, to be repeated, was ordered, to move the bowels This was all rejected.

I now discovered, for the first time, and this was on the evening of the second day of my attendance, that there was some tenderness over the abdomen, on pressure—and some fever, though not very great. Thinking there might be some degree of inflammation present, and that it was not impossible for colic and inflammation to be combined, I determined to draw blood, and took about two ounces, and then had a large blister applied over the region of the stomach and bowels. She was left with an anodyne for the night.

Saw her next morning. No better. Had vomitings in the night, and the pain continued all the time, though still returning at times with greater violence.

Being anxious that the bowels should be opened, all anodynes were stopped, and senna and salts were administered in repeated doses, four in number, without effect—two of which, however, were rejected.

*Evening.*—Stomach continues sick, and now rejects almost everything taken. There being some thirst, the free use of ice was allowed, and injections ordered. At bed-time visited her, and found the pain had increased on pressure over the region of the stomach and colon—that there was some restlessness, with a moderate degree of fever. Ordered 24 leeches to be applied upon the abdomen, and followed by poultices.

Next morning saw the patient early. Found her no better, but rather worse. Sick stomach through the night, with occasional vomiting. Pain all the time, but still greater at some moments than others. The bowels not moved yet. Ordered the warm bath and injections to be repeated while in the bath, and the following pills: ox-gall and hyoscyamus—five grains of former with two and a half of latter, and croton oil, two drops, made into four pills—to be given alternately every two hours till the bowels were moved.

Mid-day saw patient. No better. Bowels not yet moved. Some of the medicine rejected.

Professor Monkur was now requested to see the case with me. At 4 o'clock, P. M., we met. The patient no better, and no operation yet.

The doctor advised calomel to be given in large doses. Three powders were ordered; the first of twenty, the second of fifteen, and the third of twenty grains; the first to be followed in two hours with half an ounce of ol. tereb. rubbed up in an emulsion—and so on through the night, till the bowels were moved.

Next morning both sent for to see the patient early. Found her much worse, though she retained the medicine, and took the whole prescribed; which amounted to forty-five grains of calomel and one ounce oil turpentine—which, added to the twenty-two grains previously given, made now sixty-seven grains of calomel in her system—and yet no motion of the bowels. As the was no time to lose, the doctor thought we might venture on five drops of croton oil at a dose, as he said he had given as high as seven drops with good affect. This was given, injections of turpentine ordered and the patient visited again at 10 o'clock. Found the medicine had been retained, but bowels not moved. She was now evidently sinking fast. There was great restlessness, tossing from side to side of the bed; pain not so great, but still complains very much. Ordered mercurial unguent to be rubbed freely over the abdomen, inner side of the thighs, and arm-pits—and directed a dose of oil.

6 o'clock, P. M.—Met, and found there was still no operation, and that the patient was evidently *in articulo mortis*. She died about three hours after.

*Post-mortem Examination*, 10 *o'clock next morning*.—Abdomen considerably distended. Colon greatly enlarged by wind, fluid injections, and some feculent matter. No hardened fæces discovered. Next to its size, the most prominent alteration was the high grade of inflammation seen throughout its whole course, and most especially on its left ascending portion, commencing at the caput coli. Here the redness was intense, with incipient patches of mortification at different points. The small intestines showed a considerable amount of redness in different parts of their course, but not in so high a degree as the colon. The colon, in fact, showed that it was the great focus of all the distress, and the cause of death. No hardened and impacted fæces, no intussusception, no strangulation, could be discovered—the inflammation alone seeming to be the cause of all the torpor and want of contractile power shown by the bowels. The distention of the colon by wind or gas may, probably, have had some share in this general paralysis of its muscular apparatus. The stomach was slightly inflamed—also the peritoneum. Liver and spleen looked healthy. Uterus somewhat inflamed—and the right Fallopian tube, with its fimbriated extremity, greatly engorged with venous blood.

*Remarks*.—It seems evident, at least to our mind, that in the above case there must have been inflammation of the colon from the very beginning, and that its true character was masked by the symptoms of colic, which were associated with it, and predominant at the outset. And this teaches us the important fact, that not only these two diseases can come together, but that an insiduous, highly dangerous and fatal inflammation may also be going on at the same time in the system, unsuspected, till the Rubicon has been passed, the citadel of life stormed, and medical skill consequently put at defiance. Most respectfully,

W. R. HANDY.

BALTIMORE, Md., Oct. 18, 1847.

We think, with the writer, that colonitis existed from the beginning; and, we would add, that the case teaches the importance of avoiding powerful cathartic remedies under like circumstances. Leeches to the abdomen or anus, fomentations or cataplasms, and opium in decisive doses, would, in our opinion, have been far more likely to have obviated constipation by relieving its proximate cause, viz., inflammation. As connected with the use of cathartics, the case is highly instructive.—*Ed. Buff. Med. Jour.*

